# Negotiating multisectoral evidence: a qualitative study of knowledge exchange at the intersection of transport and public health

**DOI:** 10.1186/s12889-016-3940-x

**Published:** 2017-01-05

**Authors:** Cornelia Guell, Roger Mackett, David Ogilvie

**Affiliations:** 1 MRC Epidemiology Unit and UKCRC Centre for Diet and Activity Research (CEDAR), University of Cambridge School of Clinical Medicine, Box 285, Cambridge Biomedical Campus, Cambridge, CB2 0QQ UK; 2 Department of Civil, Environmental and Geomatic Engineering, University College London, Chadwick Building, Gower Street, London, WC1E 6BT UK

**Keywords:** Multisectorality, Knowledge exchange, Evidence based practice, Public health, Population health interventions, Transport, Ethnographic research, United Kingdom

## Abstract

**Background:**

For the prevention and control of chronic diseases, two strategies are frequently highlighted: that public health should be evidence based, and that it should develop a multisectoral approach. At the end of a natural experimental study of the health impacts of new transport infrastructure, we took the opportunity of a knowledge exchange forum to explore how stakeholders assessed, negotiated and intended to apply multisectoral evidence in policy and practice at the intersection of transport and health. We aimed to better understand the challenges they faced in knowledge exchange, as well as their everyday experiences with working in multisectoral remits.

**Methods:**

In 2015, we conducted participant observation during an interactive event with 41 stakeholders from national and local government, the third sector and academia in Cambridge, UK. Formal and informal interactions between stakeholders were recorded in observational field notes. We also conducted 18 semistructured interviews reflecting on the event and on knowledge exchange in general.

**Results:**

We found that stakeholders negotiated a variety of challenges. First, stakeholders had to negotiate relatively new formal and informal multisectoral remits; and how to reconcile the differing expectations of transport specialists, who tended to emphasise the importance of precedence in guiding action, and health specialists’ concern for the rigour and synthesis of research evidence. Second, research in this field involved complex study designs, and often produced evidence with uncertain transferability to other settings. Third, health outcomes of transport schemes had political traction and were used strategically but not easily translated into cost-benefit ratios. Finally, knowledge exchange meant multiple directions of influence. Stakeholders were concerned that researchers did not always have skills to translate their findings into understandable evidence, and some stakeholders would welcome opportunities to influence research agendas.

**Conclusions:**

This case study of stakeholders’ experiences indicates that multisectoral research, practice and policymaking requires the ability and capacity to locate, understand and communicate complex evidence from a variety of disciplines, and integrate different types of evidence into clear business cases beyond sectoral boundaries.

**Electronic supplementary material:**

The online version of this article (doi:10.1186/s12889-016-3940-x) contains supplementary material, which is available to authorized users.

## Background

Chronic noncommunicable diseases are the leading cause of mortality worldwide and a central public health challenge [1]. For their prevention and control, a multisectoral “whole-of-government” or “health in all policies” approach is explicitly called for, for example as set out in the *WHO Action Plan 2013–2020* [2], and should include sectors beyond health such as education, employment, finance, industry, environment and energy, to name a few. In England, public health was brought back from the National Health Service into local government in 2013 to facilitate cross-remit working, and policy documents such as *Everybody Active, Every Day* have made a case for addressing physical inactivity across society and in all domains [3].

A second major current thrust in public health is that policymakers and practitioners are increasingly challenged to be guided by evidence based on research [4, 5]. Health researchers, in turn, are tasked by funding bodies to demonstrate positive social and economic impact [6]. While “health in all remits” is still surprisingly under-researched, there is a large body of research within and outside the health field to investigate how evidence-based policy and practice might best be achieved. Over the years, conceptualisations have moved from linear models of “knowledge transfer” to the more discursive notions of “knowledge translation” and “knowledge exchange” that assume a certain degree of dialogue and co-production of knowledge [7, 8]. Academics have interrogated the understandings of “knowledge” by disciplines, individuals and institutions, and distinguished between data, information and evidence. “Knowledge” in knowledge exchange can be individual or collective, explicit or tacit, context-bound, socially shared, and value-laden [9]. However, what is known about knowledge exchange largely applies to individual knowledge (to inform clinical practice), and research has only recently turned to understanding collective, organisational processes [10]. It can borrow from research on the haphazard, chaotic and incidental nature, the “social drama” [11], of institutional decision-making [12, 13].

Despite all this research activity, there appears to be limited evidence for the effectiveness of “evidence-based knowledge exchange” and its underlying mechanisms, and little progress in effecting (or at least evaluating) successful knowledge exchange [10, 14]. One possible explanation could be that many studies have shown little understanding of the “realpolitik of ‘getting evidence into practice’” [9], and some academics argue that what is needed is to understand the context and complexity of evidence use in detail, as “research is unlikely to provide context-independent evidence” [10, 15]. Multisectoral evidence – produced by a variety of disciplines with multiple methods and research designs – is discussed in similar ways. While there might be established ways of judging evidence for its rigour and strength within health and medicine, an increasingly outwardly looking field that embraces evidence from other sectors and disciplines has a much more challenging task at hand. Nonetheless, it is urged to neither abandon the notion of evidence review and appraisal, nor dismiss solutions for which evidence is less easily appraised [16]. On the contrary, public health strategies, which cut across sectors such as urban planning, agriculture and international trade, must get to grips with the type of evidence that is produced within these sectors, and with novel research methodologies such as natural experimental studies [17].

This qualitative study explored knowledge exchange in the context of multisectoral public health, at the intersection of health and transport [18]. Encouraging “active travel” on foot or by bicycle by improving infrastructure such as cycle routes might be a “best buy” for increasing physical activity levels within populations [3]. We had the opportunity to investigate a natural experimental evaluation of new transport infrastructure and its potential impact on active travel with a pool of stakeholders who work across both sectors. The aim of this qualitative case study was to understand how multisectoral evidence is assessed and negotiated by stakeholders at the intersection of the health and transport fields. We used the method of ethnography, which combines observations and interviewing to “study social interactions, behaviours, and perceptions that occur within groups, teams, organisations, and communities” [19].

## Methods

### Study design and participants

We used a case study design, aimed at exploring and capturing the complexity of a single case [20]. Our case, the Commuting and Health in Cambridge study (henceforth, the “busway study”), had investigated the magnitude, nature and population distribution of changes in the travel behaviour, physical activity and related health indicators of commuters who travelled to work in Cambridge associated with the opening of a new guided busway system in 2011 [21]. The guided busway – similar to a tramway – offered a new bus service on a segregated track along with a new off-road walking and cycle path. Data collection had finished in October 2012, producing over twenty scientific publications, using a variety of quantitative and qualitative, epidemiological and social science research methods.

In January 2015, the end of this multi-disciplinary, multi-method research project was marked by a stakeholder forum to present and discuss its findings, and this provided the opportunity to explore the process of knowledge exchange “in practice”. We decided to use ethnographic methods, participant observation in combination with semistructured interviews, which allow the collection of data about a phenomenon (knowledge exchange) as well as people’s accounts of the phenomenon [22]. The interactive event enabled us to record public discussions and knowledge exchange interactions between researchers and their stakeholders, and in the interviews we could invite more in-depth reflections on this particular knowledge exchange event and people’s experiences more generally.

The stakeholder forum had been planned independently of this knowledge exchange case study. The format was intended to encourage dialogue, interaction and exchange in relation to the research findings, and associated research and practice topics and interests, between the study team and invited stakeholders. The forum began with an overview of the busway evaluation and its key findings. Attendees then were able to circulate around a number of stands in an “open day” or “market place” format at which they could discuss relevant methods, findings and topics with members of the research team and other attendees. The forum closed with a plenary session to identify some key learning points from the forum and a commentary on their implications for policy and practice.

The forum was attended by 41 researchers, practitioners and policymakers. They worked at the intersection of transport, health and related areas such as sustainability or urban planning. Many had experience in several sectors and remits. Most were long term stakeholders of the research institution and received regular communications from it such as newsletters or invitations to events. Some were directly involved in the busway that had been evaluated. Seventeen stakeholders agreed to be interviewed in three pilot interviews and/or fifteen follow-up interviews. They belonged to a group of 21 directly contacted stakeholders who had expressed an interest during the forum or communication about it, and did not work too closely with the study team. They identified themselves as researchers (*n* = 4), practitioners or policymakers in national (*n* = 2) or local government (*n* = 9), and representatives from the third sector (*n* = 2), working on cross-sector topics but identifying themselves as based in the transport (9) or health (8) sectors. Two stakeholders were interviewed but could not attend the forum. The case study received approval from the University of Cambridge Psychology Research Ethics Committee (Pre.2014.97). All participants gave their informed consent, for the participant observation during the workshop by giving their consent online at the point of registration, and on separate consent forms prior to each interview.

### Data collection

During the forum we conducted participant observation to capture interactions, and used these as a starting point for reflections and eliciting contextual information in semistructured interviews with a subset of the attendees. For the participant observation, an experienced ethnographic researcher (CG) walked between the market place stands, observed the panel discussion and engaged in conversations in breaks and during lunch. She paid particular attention to discussions about the relevance and usefulness of evidence; to expressed interests in or preference for different types of evidence; and to informal conversations between stakeholders, and between stakeholders and the study team, about any concerns, applications and adaptations relating to the use of evidence in their specific contexts. The participant observation of the event was known to the attendees at the outset, and the researcher was introduced at the beginning of the event; the researcher followed up on some interactions or discussions in direct conversations with particular stakeholders. Observations and conversations were recorded in ethnographic field notes. Nine busway study team members and forum facilitators (including two experienced ethnographers) also wrote short reflective summary field notes, in particular about “stand-out” interactions, queries or contributions they experienced during the event. The participant observation served as a preliminary data collection phase to inform the guide for and analysis of the semistructured interviews.

Several months before the forum, we conducted three semistructured pilot interviews in preparation for the event and to pilot the initial interview guide. As the participant observations of a one-off forum could capture only partial insights, we used the follow-up interviews to investigate any emerging issues in greater depth in a one-to-one setting, and explored these within the particular context and experience of each interviewee. The forum format and discussions during the forum served as a prompt at the start of each interview (see interview guide in Additional file 1). We asked about stakeholders’ assessment of the evidence presented to them, and explored their experience of using evidence and encountering resistance to this, for example regarding particular types of evidence and methods of communication. The interviews were scheduled shortly after the forum to give the participants some time to reflect on possible plans for using the evidence. Ten of the follow-up interviews were conducted within 1 to 4 weeks after the forum, and another five within the next 4 weeks. The interviews lasted about 30 min and were mostly conducted by phone, or sometimes in person (at either the participant’s workplace or the research institution) according to the convenience of each participant. The interviews were audio-recorded with the participants’ permission and transcribed verbatim.

### Data analysis

We conducted a thematic analysis of field notes and interview transcripts with a pragmatic coding framework guided by our research questions [20]. We retained some flexibility for inductive analysis of emerging unanticipated issues. The field notes were analysed immediately after the stakeholder forum to inform the guide for the follow-up interviews. The transcripts were analysed to build on the initial coding and emerging themes. CG led the analysis and did most of the coding; DO independently coded three sample transcripts to challenge, complement or confirm analytical interpretations. Constant comparison between themes that emerged from coding, and between the multiple sources of field notes and the interview transcripts, also helped to triangulate our findings. All analysis was arrived at iteratively and in consensus with the authors. The draft manuscript was also shared with selected participants for “member-checking” [23]. Analysis was aided by the qualitative data analysis software NVivo 10 [24].

## Results

### Negotiating sector-specific evidence in new multisectoral roles

The roles and remits of many of our study participants were multisectoral, either by definition (being assigned to a different sector or tasked to work across sectors) or by dedication (having interests that spanned sectors). Participants were either public health researchers, practitioners or policymakers who integrated travel and transport into health cases; or transport researchers, practitioners or policymakers who considered the integration of health benefits into the business cases or planning of transport schemes. As explained earlier, such multisectoral roles had recently been formalised and institutionalised as the government had (re-)integrated the public health function from the National Health Service to local government in England. However, participants from public health backgrounds, who found themselves embedded in this way into existing structures of local politics and policymaking, explained that even within this new policy environment of shared priorities, individual effort was still required to breach working in silos. And existing roles in other sectors required a new (and less formal) openness to allowing health into their remit.

Even shared interests required to be allocated to specific budgets; designated “shared priorities budgets” were considered very limited. Public health policymakers explained that in the absence of shared budgets, a “health benefit” business case was often too readily considered a clear responsibility for the health budget. At the same time, they saw a danger in considering health as a pure “add-on” to other priorities and benefits such as economic growth, because “add-ons” were vulnerable in a time of financial austerity and budget cuts.

“Because ideally what you want is the person in transport to actually have enough appreciation of public health that that gets borne in mind in the interventions they put in, whereas it’s almost a bit of an add-on. […] And […] the add-ons are always the first things to be cut when budgets are squeezed…” (SE12; research, health)

During the forum, a heated debate focused on the challenge that sharing priorities and budgets also meant co-developing business cases, despite “the use of different language” in health and transport. This was not so much about sector-specific language – all were familiar with the terminology surrounding active travel, for example – but about different expectations of what was considered relevant evidence. Participants described this as a “culture shock” that inexperienced public health trainees, in particular, might experience when entering the world of local policymaking. The consensus was that health colleagues were very used to the notion of evidence synthesis and the status accorded to evidence from randomised controlled trials (RCTs), guided by the notion of an “evidence hierarchy”. In contrast, transport colleagues were more interested in precedence – whether something had been done before and “worked” elsewhere.

“…this is a bit of a generalisation here, but in general public health people would love to see […] an effectiveness study with a low *p* value and a good return on investment, and a high impact, […] but transport people would like to hear a good story and know that it’s […] working”. (SE02; research, health)

While this divergence between evidence synthesis and precedence was largely explained by different disciplinary and training backgrounds, the potential tension between “hard facts” and “good examples” was often reconciled in practice. To make a convincing argument when addressing and lobbying politicians and decision-makers, a third translational effort was described, connecting personalised examples with snappy cost-benefit statistics. While business cases would benefit from convincing statistics demonstrating effectiveness, if a scheme “worked” or “paid off” across sectors, participants felt that elected members liked to see such numbers translated into a relatable illustration.

“[…] there’s a need for key headline quantitative stuff, so like charging for parking we reduced car use by 20% which in turn increased active travel by 60%, […] which is all great […]. But at the same time we need those stories as well because they come to be the ones that people really listen to and can relate to, and certainly in the political environment that we’re working in, the members would much rather hear ‘Barry from Fakenham […] said […] he’s now walking five times a week and he would never have done this unless they started charging for parking’”. (SE03; local government, health)

### Negotiating complex evidence derived from novel research designs

Participants’ descriptions of priorities and preferences, which related to evidence synthesis and precedence, did not, however, map onto neat juxtapositions in their experience with research evidence. Evidence in this field is often derived from evaluating complex population health interventions [17]. In the busway study, this entailed evaluating the effects of infrastructural changes that had the potential to encourage active travel. Structural changes of this kind affect and are shaped by both the physical (built) and social environment, and are therefore inherently contextual and complex. Reflecting on the study findings presented during the stakeholder forum, participants described the different aspects of assessing this kind of evidence and their efforts to negotiate challenging research findings.

Participants were very aware that the evidence produced in this particular study was inherently tied to its setting, Cambridge. In this university city, cycling is relatively common across all ages and backgrounds and compared to the rest of the country [25], and the new guided busway is a relatively rare form of transport infrastructure around the world. Despite a general favouring of precedence, participants from the transport sector in particular were aware that infrastructural changes were closely shaped by the local context in which they were implemented and would not necessarily work elsewhere.

“…one calculation I want somebody to make is how much of the new development around the city would be able to take place in another city where there isn’t a strong cycling culture […]. So you know much of the development in, as I say, that’s able to take place around Cambridge, in another city possibly wouldn’t be able to because they wouldn’t be viable in transport terms…” (SE08; local authority, transport)

Closely related to this challenge of generalisability, participants also understood that evidence was rarely clear-cut when derived from natural experimental studies aiming to evaluate large-scale structural changes, the outcomes of which are difficult to measure, let alone predict. Transport specialists were perhaps more at ease with ambiguous evidence, as they were less experienced with working with clear-cut evidence from RCTs, as described above. However, those working in the health field were not necessarily better equipped to critically appraise the quality and strength of evidence that was not produced through RCTs.

During the forum discussion as much as during the interviews, there were calls for knowledge exchange to include translation of how the evidence was produced, not merely what it meant. Participants said that colleagues often lacked the necessary skills to assess evidence and how it was produced; or to understand how evidence about other interesting and relevant issues, such as the monetary value of particular interventions, might be difficult to produce.

“… if you take an RCT about an intervention clearly it’s incredibly important and is very, very clear, but most research is not 100% or anywhere near that to be honest”. (SE12; national, health)

“The difficulty is as soon as people have to […] critically appraise things themselves for what’s in it for them you’re going to lose a lot of people because they don’t have the skills or the time or the inclination to do it”. (SE11; research, health)

A case for this was the presentation of evidence from the busway study about the potential effect of charging for car parking on promoting the integration of more active travel into people’s commuting journeys. Understanding of the evidence that was presented was variable, but it was received overwhelmingly positively by the participants as clear-cut evidence showing unambiguous evidence that parking fees could effectively deter people from commuting by car. This sparked much debate about the seductive simplicity, but also the political contentiousness, of seeking to influence behaviour in this way. The heated discussion and subsequent interview responses included references to the need for action if presented with such convincing and (technically) easily-implemented evidence, but also warnings that any such scheme could easily be derailed if it were seen to oppose the political or economic mainstream that worried more about the acceptability of the measures to the electorate. From a research perspective, however, the challenge was different. The researchers who presented the findings on parking charges considered the evidence to be relatively weak. The observational epidemiological research design had indicated only evidence of associations, and therefore of the *potential* to influence commuting behaviour through parking charges that warranted further intervention studies to establish a causal relationship – a nuance of research design that seemed less apparent, or less important, to many participants from policy and practice.

### Negotiating partial and strategic evidence

While the transferability of research findings was a concern, some participants pointed out that decision-making in policy and practice was routinely based on such partial, incomplete evidence.

“…policymakers make decisions on the basis of incomplete evidence because if you waited for complete evidence you’d never make a decision; so there’s this trade-off […]. So it would be good if the debate wasn’t centred on sufficiency of evidence where […] policymakers say ‘does it work or doesn’t it work?’ and the researcher [...] saying ‘well, it’s not as simple as that’ because then it’s a dead debate because it doesn’t take us anywhere”. (SE04; third sector, transport)

Participants from all sectors discussed this challenge of “not knowing enough” as a common, manageable experience for decision-makers but also as a weakness for lobbying them convincingly. From a research perspective, this was debated in terms of a shift from “evidence-based” to “evidence-informed” practice in public health. This was echoed by suggestions that while practitioners and policymakers might be quite experienced and familiar with the notion of “evidence-informed” practice, researchers might need to be more confident in conveying the “best available” evidence rather than highlighting remaining scientific uncertainties that might delay action.

“I’ve started using ‘evidence-informed’ recently. […] you want the scientific evidence but then you’ve also got other sorts of evidence that politicians or policymakers […] will take into account […], and also not all evidence agrees with each other, you do ten studies, they don’t come out with a nice clear answer, […] and actually that doesn’t really matter because what you need then is someone with the judgement to pull those together, use that to inform their decision”. (SE12; research, health)

Participants also described their efforts to use evidence strategically. Their comments in both the open forum discussion and individual interviews described a tension when making a case for “health” as a strategic and convincing piece of an argument. Epidemiological evidence for the health benefits of physical activity had political traction, but concrete evidence for the effects of particular interventions – that infrastructure A leads to health outcome B – was hard to come by.

“I always try to say that health […] is a very strong lever to use when we want to talk about active travel. So if I can supply the data that says, if so many more people could cycle or walk, then we’d have so many fewer heart attacks, which means so many fewer admissions, or so many fewer sick leave days, or something. That’s very powerful”. (SE05; local government, health)

On the one hand, a health argument was considered to carry weight when addressing decision-makers, and epidemiological evidence could even be used as proxy cost-benefit evidence because a healthier population or workforce would surely mean lower healthcare costs or more productivity. On the other hand, policymakers and practitioners expressed their frustration that a clear monetary value could often not be attached to a specific infrastructural intervention – in particular, not to the case study at hand – and that this could weaken the argument. Public health was often seen as a “long game”, involving health outcomes that might range from the prevention of chronic disease and early death to more immediate but less quantifiable outcomes such as reduced absenteeism or better mental wellbeing. While they understood that a complex infrastructural change might result in health co-benefits that took a long time to emerge and were difficult to measure or convert to a monetary value, some regretted the lack of an explicit health economics element in the busway study.

“…monetising the costs to the healthcare sector of other policies, so if we make this change to the transport system it will benefits their healthcare system to the tune of X million Pounds is something that some people are really uncomfortable with doing because obviously it’s quite a flawed and simplistic thing to do, but unfortunately if you want to change decision makers’ minds you do need to put it in a way that they understand […]”. (SE06; local government, health)

### Negotiating the production of multisectoral evidence

Finally, participants were all not only receivers of knowledge exchange exercises but – true to the notion of “exchange” – producers of such efforts. Many of our participants not only operated at the intersection of different sectors, and often disciplines, but also had experience in research, practice and/or policymaking, having moved between different professional roles in the course of their careers. Understandings about the direction of influence were echoed by many, but also contested.

Most participants agreed that it was very important that research spoke effectively and relevantly to issues in policy and practice. However, they recognised that not all researchers might be interested in communicating their research, and that not all policymakers and practitioners had the resources, capability or political will to base their advice and actions on scientific evidence. Knowledge brokers and generalists could help bridge this gap, and some participants attempted to fulfil this role themselves, based on their work across remits.

“So the difficulty is both sides, it’s not just policymakers ignoring evidence or not understanding evidence, it’s researchers translating evidence into usable decision, actionable things”. (SE04; third sector, transport)

“I work a lot supervising public health registrars and they have to do a lot of work to simplify their message down to a level where it will actually be engaging and compelling and easy to understand, and I have to do a lot of work with them to keep rewriting the same thing over and over until they get it simple enough that someone [understands] who was in a hurry”. (SE06; local government, health)

The other direction of knowledge exchange, heatedly debated both in front of colleagues and anonymously during interviews, was the direction from policy and practice to research. Some suggested that for research to achieve impact, perhaps the policy and practice sectors should have more influence on setting research agendas. Some participants would have liked to see researchers involving policymakers and practitioners from the start of planning new research projects, and that this required effort from both sides and openness to share ideas and plans with each other.

“We’ve got practice going along, we’ve got research saying this is better, but […] they’re just not coming together. […] I mean, research as well […] needs to know what’s coming up, so that they can start working towards it, needs to know what local priorities are. […] But it’s difficult […], I’ve asked for forewarning of potential projects […] and I just get diverted to a website that is publicly available. That’s not helpful. […] People are very scared of sharing and exchanging knowledge, and I think it’s detrimental in the long term”. (SE06; local government, health)

Others cautioned that evidence produced by research organisations would need to be seen as independent; otherwise it would lose its power, authority and impact.

“I’m not sure that I think, you know, practitioners should be directing the agenda of academia. That would concern me a bit I think, you know. I think academia should be a very much an independent force…” (SE14; research, transport)

This question of the level of involvement in research, and whether there might be a limit to such involvement, seemed to depend on the topic matter and context, and was perhaps most importantly felt in regard to politically charged and highly polarised topics. The importance of assumed “stakes” was raised in relation to contentious schemes such as car parking charges, but some participants felt that any infrastructural “anti- or non-car” scheme would be better supported by independent academic evidence than by third sector advocacy. Others pointed out that evidence was never neutral, and research was certainly not ideology-free.

“[…] ideology informing the research that’s done and […] it always has and always will. I think the best that we can do is be open about it and clear about the drivers behind it and understand that this is going to govern the research questions that we ask […]. So I don’t subscribe to the idea that there’s, you know, we’re building an objective body of knowledge and evidence which policymakers should adopt…” (SE04; third sector, transport)

## Discussion

### Summary of findings

In this ethnographic case study, participants had experienced knowledge exchange in their daily practice as a variety of challenges, tensions and negotiations. Their new roles in the multisectoral field of transport and health varied in formality and priorities which had to be negotiated within and between sectors. Language and expectations of the type of evidence differed and created similar challenges. The most pronounced difference was that between what was described as a preference for precedence by transport specialists, and for systematic evidence synthesis by public health specialists. However, evidence was experienced as contextual, complex, sometimes conflicting and often incomplete. Complex research designs produced partial evidence, but participants suggested that decision-makers were used to making decisions despite such uncertainties. That said, evidence strength could be rated in different ways, sometimes shaped by the ability to “read” evidence correctly, and even seemingly clear-cut evidence could be politically contentious. In addition, the potential health co-benefits of transport schemes were appreciated to have traction in arguments made to decision-makers, but were not necessarily easily investigated by researchers or translated into convincing cost-benefit analyses. Finally, knowledge exchange happened within a context of multiple directions of influence and expectations thereof. Participants discussed that researchers needed the skills to make their evidence understandable, and that some might not be interested or well suited to undertake this task themselves. Instead, knowledge brokers might be better placed to do such translational work. Finally, policymakers and practitioners considered the benefits, but also the potential disbenefits, of influencing research agendas more directly.

### Results in context

The findings echo much of the knowledge exchange literature, such as the necessity of translating knowledge into different sectoral and disciplinary languages and having the time and skills to do so [8, 15, 26, 27], appreciating different types of knowledge or a combination thereof as useful, convincing or strong [28–30], and the ways in which evidence can be used strategically to serve particular outcomes [13, 30]. While it might not be a particularly new insight that different sectors inhabit different epistemological worlds and appraise knowledge differently, the experience of practitioners and policymakers whose remit spans several sectors has rarely been captured. Two further issues are selected here as particularly pertinent to this specific context: the role of uncertain evidence in knowledge exchange, and the political nature of evidence.

This case study of evidence derived from a natural experimental evaluation represents the challenges of much public health research. Interventions of this kind outside the health sector can create a natural opportunity to produce new learning about how best to produce beneficial outcomes across the health and transport sectors. However, evaluating the impact of complex interplays of social, environmental and political-economic contexts on population health behaviour produces complex evidence that can rarely be distilled into simple, definite answers [17]. Rather it forms a jigsaw of partial evidence derived from a range of methods, that might gradually be built up across multiple studies over time to form an emerging, more generalisable picture [28]. Researchers who produce this evidence can accept such uncertainty in their daily practice, relying on tools such as confidence intervals and sensitivity analyses to communicate levels of uncertainty, and suggesting further research and refinement of scientific concepts and methods. Practitioners and policymakers, however, face the dilemma of being tasked to put such partial evidence, and from sectors they might be less familiar with, into comprehensive action, eradicating uncertainty from business cases and policy documents in the process.

Lipsky’s seminal work on “street level bureaucracy” was one of the first that aimed to understand individuals’ efforts to implement organisational decision-making with its inherent uncertainties [31]. He found that front-line public service workers had to negotiate multiple, interacting factors such as regulations, policy directives and expectations of elected officials which were not necessarily aligned. This resulted in discretions and inconsistencies in putting policy into practice. More recent work on evidence based policymaking has explored ethnographically how civil servants need to craft “persuasive policy stories” from a large but inconclusive amount of evidence [32]. Similarly, a study on health policy has traced how varying evidence – in this case on screening for a range of cancers – and its context resulted in different appraisals and use of evidence, and different policy recommendations, by different expert groups [33].

Part of the uncertainty is that evidence is not always understandable and may be produced through methods that are novel, specialised or complex. Studies from the field of environmental public policy, for example, have described how practitioners and policymakers have to grapple with new research technologies such as modelling, and those skilled to read this evidence are better placed to influence policy outcomes [34]. In our study, researchers, practitioners and policymakers described that they needed to understand a range of evidence produced from multiple methods and disciplines, and negotiate what constituted relevant, reliable or relatable evidence across sectors. This varied, complex and challenging evidence formed part of their everyday practice – echoing research that suggests that evidence jigsaws can be helpful in making a policy case [28] – but also hindered their ability to influence decision-makers with clear advice. It has been suggested that perhaps what policymakers need is not more evidence, but more systematic “methods for identifying, interpreting, and applying evidence in different decision-making contexts” [33].

Also, complex population health research does not produce evidence to influence individual clinical decisions, but aims to effect population-level responses in policy and practice and is therefore more exposed to political and public scrutiny [35]. This is particularly pronounced in the “more overtly politicised local government space” into which public health has recently moved in England [30]. Importantly, it is not just actors and spaces that are political; evidence is itself far from neutral and can be positioned in varied ways in policy and practice, thereby adding to uncertainties surrounding evidence. Tension between the assumed objectivity of research and subjectivity of politics arise if decision-makers want the evidence to confirm that the chosen outcome from a decision is correct, or conversely if the evidence points to politically or socially contentious actions. Our participants, for example, explained that infrastructural schemes were highly politicised and that in the current climate of financial austerity in the public sector, any business cases were increasingly reduced to an economic bottom line. It has been suggested that evidence in policy should be considered as “socially embedded in authority relations” [36], and that politics is not a third pillar besides research and policy but runs through all domains [30].

While much of the knowledge exchange literature has focused on the practice or policy side, it is equally important to note that evidence production – research – is also not free of ideology and politics [11]. Some of our participants felt strongly that research should be guided by policy agendas, and that research and practice should be developed simultaneously and iteratively for more policy-relevant evidence, better informed practice, and timely evaluation. Others were concerned that research ought to retain its public image of neutrality, and thus retain its authority or power to influence policy agendas. This sentiment, of course, also pointed to the political nature of research.

### Implications for practice and future research

More research about the uncertain and political nature of evidence is clearly still needed as these play out in different public health contexts in research, policy and practice. While this case study particularly focused on the context of active travel and transport as a target of population health intervention, it indicates that through the lens of a particular context of transport and health, larger barriers to knowledge exchange in multisectoral, evidence-based public health can be identified [35, 37]. Most public health remits span sectors, fields, remits and disciplines, whether by interest or institutionalised [38]. We need to learn more about the realpolitik and contextual factors of multisectoral collaboration [39], which are meant to underpin current public health policymaking, for example the ways in which partnerships between health, agriculture and national and global industry might or might not work to ensure the accessibility and affordability of healthy foods [40]. Such analysis of institutional knowledge processes could help to understand how multisectoral working can be successful, for example how to achieve integration of priorities and partners in multisectoral collaboration [38]. Moreover, multisectoral partnerships can be just as polarising and politicised as evidence, and this can affect how evidence can be used and abused strategically. In-depth policy analyses could, in fact, focus on a range of public health strategies such as the taxation of sugar sweetened beverages and other unhealthy foods [41], understanding the broader perspective of “actor networks” that hold influence and authority over decision making as well as micro- or “street-level” negotiation. This could inform strategies for engaging effectively with industry opposition and political and public scepticism.

### Strengths and limitations

We believe the strength of the research design was its case study approach; using a “real life” example of knowledge exchange attached to a particular research project enabled us to understand the contextual factors that shaped, supported and hindered the use of evidence by our stakeholders [32, 42]. Exploring a particular, poignant context helped to focus our debate with them and encourage reflection. The case also helped to highlight particular topics, such as polarising evidence and evidence from complex research designs. Moreover, using an ethnographic method that combined participant observation with interviews strengthened our analysis through the triangulation of themes and discussion. We were able to record formal and informal exchanges between stakeholders and between “them” and “us”, and in follow-up interviews we could allow space for reflection on the evidence presented and on opportunities to make use of it in their daily practice. Reflecting with our stakeholders during the event and subsequent interviews, and with some of them during the analysis, we could develop “policy points” with them to summarise insights from this study (see Additional file 2).

Our research design also had limitations. We did not include much of a researcher perspective in this case study, and mostly interviewed stakeholders in policy and practice roles. This was mainly because most of the researchers who attended the forum were attached to the busway study, and were asked to write field notes for this case study. However, some of our participants from policy and practice had experiences as researchers themselves and included reflections from a research perspective. National government representation was also limited at the forum, and therefore in this study. This may have reflected the limited availability of civil servants shortly before a general election in particular, or for knowledge exchange events more generally. This study therefore included a self-selected pool of participants with a particular interest in the multisectoral subject matter and perhaps in evidence-based practice and policy and knowledge exchange more generally. Similarly, this may have limited the responses in the interviews, as those agreeing to be interviewed may have been particularly interested in the topic. While qualitative purposive samples aim for knowledgeable, “information-rich” participants [22], discussions and interviews with less informed practitioners and policymakers in the field may have yielded different or additional insights. Finally, elected representatives and members of the general public were not represented at the forum or in this case study, and this was highlighted as part of critical feedback from participants. It was also suggested that for future events the organisers could encourage invitees to “bring a friend”, or indeed to “bring an enemy”, to broaden the range of perspectives reflected in discussion and the interviews.

## Conclusion

Studies of multisectoral population health interventions, such as those aiming to encourage physical activity by changing the built environment, produce complex evidence and aim to inform practitioners and policymakers across a variety of sectors. Knowledge exchange in multisectoral public health requires people who can locate and interpret evidence from a variety of disciplines, and can find a common language to integrate such evidence clearly into business cases. Practitioners and policymakers would welcome help from researchers to translate complex, challenging and often incomplete evidence into clear recommendations, and clearer mandates from politicians for health as a priority in all remits. These would strengthen their efforts to integrate health benefits in business cases and promote multisectoral cooperation.

## Additional files

Additional file 1: Interview Guide. (DOCX 29 kb)
Additional file 2: Policy Points. (DOCX 27 kb)

## Abbreviation

RCTs: Randomised controlled trials
