# Supplementary material for: Negotiating multisectoral evidence: a qualitative study of knowledge exchange at the intersection of transport and public health
Source: BMC Public Health. 2017 Jan 5;17:17. doi: 10.1186/s12889-016-3940-x (PMC5217628; doi:10.1186/s12889-016-3940-x)
Supplement: Additional file 1: — Interview Guide. (DOCX 29 kb) [file 12889_2016_3940_MOESM1_ESM.docx]

Additional file 1: Interview Guide

| **INTERVIEW GUIDE**  **TOPIC 1: Assessing (the busway) evidence – reflections on the forum**  **What are your general thoughts about the event you took part in?**  *Prompts: useful, appropriate, worthwhile; waste of time; confusing; and why, what part, examples. What did you hope to get out of the event? And did you?*  **Which stations did you visit? Find most/least interesting/helpful/useful/surprising? What was missing?**  *Prompts: health, environment, evaluation, mapping, drawing thread together*  **What topics or type of evidence (produced by the busway study) would you regard as relevant or usable?**  *Prompts:*  *Can you give an example – in what way was this useful/not useful? Types can relate to method (qual, quant –self-report/objective), delivery format (graphs, tables, quotes), topic (health, physical activity, transport, evaluation method); mix of evidence (‘evidence jigsaw – importance of mix, or focus on particular issues)*  **What are your thoughts about the interactive format of the event?**  *Prompts: Was it appropriate, helpful, confusing etc.; and why, examples, which parts; what conversations did you have? With whom, anything surprising?*  **Have you heard about our Commuting and Health Study in other ways?**  *Prompts: other communication about the busway study; or busway in general, or related environmental/infrastructure interventions/change studies by us or others; thoughts as above: appropriate, helpful, etc. related to what kind of information*  **Any general thoughts about events such as these?**  *Prompts: available often, attend these often, motivation, typical formats, organised by whom? What do you get out of them?*  **TOPIC 2: Applying (translating) evidence**  **Are you planning to use any of our research findings in your practice/policy-making? How? Why not?**  *Prompts: Again, reflection on topics and types of evidence, or on concrete information/research outcomes; pick one or more examples to explain how this knowledge maybe used or why not usable; already used some of it;*  **How confident do you feel using this evidence now?** *Or know where to look for it??*  **Can you explain what kind of evidence (content, study design) is usable in which circumstances?**  *Prompts: more exploration of earlier question about types of evidence but can be outside Commuting and Health study e.g. statistics for planning; photos, stories for advocacy?*  **What type of evidence is generally resisted in your experience?**  *Prompts: by yourself or your field, sector, institution, purpose (e.g. making a business case)*  **In what way do you need to adapt evidence/research outcomes? Why? How?**  *Prompts: concerning ideology, scope, audience; format, type*  **Any other thoughts on knowledge translation?**  *Prompts: relating to points raised in final discussion*  **TOPIC 3: Evidence and decision-making**  **What role does evidence play in your (approach to) decision making?**  *Prompts: e.g. to back up claims, plan strategies, estimate outcomes, set objectives; sources of evidence; why, examples*  **What role does evidence play in your organisation’s approach to decision making?** *Prompts: e.g. need for evidence or resistance; see above: to back up claims, plan strategies, estimate outcomes, set objectives; who is making decisions; why, examples; what about other organisations*  **What other factors might be relevant?**  *Prompts: economic, social, personal, institutional, ideological factors; why, examples; in what way competing, complementary etc.; how to reconcile, negotiate these*  **Would you like to add anything else?**  *Prompts: including final reflections on study purpose, design*  **Finally, what label should we give your contribution?**  *Prompts: Health, transport, sustainability etc.; local authority, national government; practice/policy/research* |
| --- |
