# Supplementary material for: Negotiating multisectoral evidence: a qualitative study of knowledge exchange at the intersection of transport and public health
Source: BMC Public Health. 2017 Jan 5;17:17. doi: 10.1186/s12889-016-3940-x (PMC5217628; doi:10.1186/s12889-016-3940-x)
Supplement: Additional file 2: — Policy Points. (DOCX 27 kb) [file 12889_2016_3940_MOESM2_ESM.docx]

**Additional file 2: Policy Points**

| **Policy Points**   - Despite recognition of the importance of multisectoral collaboration, it still requires individuals’ efforts to break institutional “silo” working. - Stakeholders ask for “health in all remits” to be supported by structural changes, clearer mandates and acknowledgement that public health can contribute to achieving other policy objectives. - Population health interventions produce complex and context-dependent evidence that requires specialist knowledge to appraise. - Knowledge exchange involving such evidence should include debating the value and limitations of research designs. - Similarly, researchers should be aware that evidence may not neatly fit the complex and context-dependent conditions, expectations and relationships of the policy or practice arenas. |
| --- |
